# *FOXE1* polymorphisms and chronic exposure to nitrates in drinking water cause metabolic dysfunction, thyroid abnormalities, and genotoxic damage in women

**DOI:** 10.1590/1678-4685-GMB-2021-0020

**Published:** 2021-10-04

**Authors:** Diana Dennys Gandarilla-Esparza, Esperanza Yasmin Calleros-Rincón, Hortensia Moreno Macias, María Fernanda González-Delgado, Gonzalo García Vargas, Jaime Duarte Sustaita, Alberto González-Zamora, Efraín Ríos-Sánchez, Rebeca Pérez-Morales

**Affiliations:** 1 Universidad Juárez del Estado de Durango, Facultad de Ciencias Químicas, Laboratorio de Biología Celular y Molecular, Gómez Palacio, Durango, México.; 2 Universidad Autónoma Metropolitana, División CSH de la Unidad Iztapalapa, Departamento de Economía, Vicentina, Ciudad de México, México.; 3 Universidad Juárez del Estado de Durango, Facultad de Ciencias de la Salud, Departamento de Investigación. Gómez Palacio, Durango, México.; 4 Universidad Juárez del Estado de Durango, Facultad de Ciencias Biológicas, Laboratorio de Biología Evolutiva, Gómez Palacio, Durango, México.

**Keywords:** *FOXE1* polymorphisms, nitrate exposure, subclinical hypothyroidism, methemoglobin, genotoxic damage

## Abstract

Nitrates in drinking water has been associated to adverse health effects, including changes in glucose and lipid levels, thyroid hormone imbalance and adverse reproductive effects. We analyzed metabolic and thyroid hormone alterations and genotoxic damage in women with chronic exposure to nitrates in drinking water. The concentration of nitrates in drinking water was quantified and according to this parameter, participants were divided into three exposure scenarios. Blood and urine samples were collected from 420 women living in Durango, Mexico and biomarkers were determined. We found nitrates concentrations in drinking water above the permissible limit (>50 mg/L), and an increase in the percentage of methemoglobin (p=0.0001), nitrite in blood plasma and urine (p=0.0001), glucose (p=0.0001), total cholesterol (p=0.001), LDL (p=0.001) and triglycerides (p=0.0001). We also found alterations in TSH (p=0.01), fT3 (p=0.0003), T4T (p=0.01) and fT4 (p=0.0004) hormones. Frequency of subclinical hypothyroidism was 8.33%; differences in *FOXE1* (rs965513, rs1867277) genotypes distribution were found and both polymorphisms were associated with a decrease in TSH. A high percentage of micronucleus in binucleate lymphocyte cells was found (35%, p=0.0001). In conclusion, the chronic exposure to nitrates in water for human consumption caused metabolic and hormonal alterations and genotoxic damage in women.

## Introduction

Nitrate is a natural compound present in soil, air and water, which forms part of the nitrogen cycle. Nitrogen and its stable forms are essential components of many molecules; however, it is also considered a potential health hazard due to the generation of N-nitroso compounds, which are pro-oxidants of several biomolecules. The pollution caused by nitrates is mainly attributed to certain industrial activities, as well as to the use of fertilizers in agriculture. Intake of nitrates among the human population occurs mainly through consumption of vegetables and processed foods, in addition to contaminated water. Water contamination occurs through leaching due the excessive use of fertilizers and pesticides that pollute the soil, which later reach the aquifers, contaminating the water that supplies the network for public use (Ombreta et al., 2018).

The Mexican Official Guidelines (NOM-127-SSA-1-1994) (http://www.salud.gob.mx/unidades/cdi/nom/127ssa14.html) established a maximum permissible limit of nitrates for human use and consumption of up to 44.3 mg/L (10 mg/L Nitrate-Nitrogen), consistent with the limit specified by the US Environmental Protection Agency (EPA). The World Health Organization (WHO) set a recommended limit of 50 mg/L (11.3 mg/L of Nitrate-Nitrogen) (http://www.who.int/water_sanitation_health/water-quality/guidelines/en/).

Upon ingestion, nitrate begins its transformation in the oral cavity by nitrate-reducing bacteria (nitrate reductases enzymes), which convert nitrate to nitrite. It is then absorbed in the small intestine by active transport, and another fraction is biotransformed by the microbiota and the acidic environment of the gastrointestinal tract, while nitrite is absorbed by diffusion across the gastric mucosa and gut wall. Approximately 5% of the nitrate ingested is reduced to nitrite by reducing bacteria present in the oral cavity, and around 25% is partially recycled in the salivary glands that concentrate the nitrate from the plasma, which is again reduced to NO_2_. After absorption, around 60% of nitrate is excreted in the urine, 3% is excreted in the form of urea or ammonium (Karwowska and Kononiuk, 2020).

During the biotransformation of nitrate, reactive nitrogen species (RNS) are generated; these are potent carcinogens for both animals and humans. The RNS generated through the nitrates metabolism include nitric dioxide, nitric oxide (NO), and peroxynitrite. These RNS can generate oxidative stress capable of damaging DNA, proteins, lipids and cell tissues, and can even compromise the function of organs such as the liver and the intragastric mucosa (Rocha et al., 2012). This compromised liver function is due to the activity of cytochrome P450 present in hepatic microsomes, which can generate nitrosamines and other compounds that are more reactive than the initial RNS (DeMartino et al., 2019). *In vitro* studies have been conducted to determine genotoxic damage from exposure to nitrate; these studies reported a higher frequency of micronuclei in individuals treated with potassium nitrate and sodium nitrate (Stivaktakis et al., 2010).

Nitrate is less toxic than nitrite because it causes the oxidation of hemoglobin to methemoglobin (metHb). Hemoglobin is responsible for the transport of oxygen through blood vessels and capillaries, while metHb is not able to release oxygen; therefore, nitrite can cause hypoxia that in severe cases may be able to cause death (Johnson et al., 1987). The consumption of water contaminated by nitrate has been associated with an increased percentage of metHb and nitrate metabolites, such as NO, which have physiological and pathophysiological effects on inflammation, vasodilation and metabolism. In other studies, it has been reported that a high NO production, possibly derived from the biotransformation of ingested nitrate, is associated with hyperlipidemia, affecting total cholesterol, high-density lipoprotein (HDL) and low-density lipoprotein (LDL) cholesterol serum concentrations; while other studies are inconclusive for whether there is an association between exposure to nitrates and serum glucose levels (Lundberg et al., 2018).

On the other hand, the thyroid gland can concentrate monovalent anions such as nitrates, leading to altered homeostasis by inhibiting the uptake of iodine, which is essential for the structure and formation of thyroid hormones. Iodine is transported by sodium/iodide Symporter (NIS), which are located in the intestine, mammary, salivary and thyroid gland and placenta (Hallinger et al., 2017). Some studies have reported that nitrates, thiocyanate and perchlorate inhibit the uptake of iodine, leading to various alterations in the levels of thyroid hormones T3 (triiodothyronine) and T4 (thyroxine), along with increased thyroid-stimulating hormone (TSH) levels (Cengiz and Bilgin, 2016). Several studies have linked the consumption of drinking water and foods high in nitrates with hypothyroidism and thyroid cancer; however, the associations are inconclusive (Donoso and Cortés, 2018).

In addition, polymorphisms in genes involved in the synthesis of thyroid hormones can also affect the circulating levels of these hormones. *FOXE1* is a transcription factor involved in the migration of thyroid precursors during the morphogenesis of the gland and subsequent cell differentiation. Polymorphisms in the *FOXE1* gene have been associated with thyroidism, increased risk of papillary thyroid cancer and sporadic follicular thyroid tumors. (Mendieta-Zerón et al., 2017; Chen and Zhang, 2018). Mutations in this gene cause Bamforth syndrome (hypothyroidism and palate abnormalities), demonstrating the importance of *FOXE1* protein in the development and differentiation of the thyroid gland. Studies carried out in European populations have shown a significant association of the *FOXE1* rs965513 polymorphism with altered levels of TSH, free T3 (fT3) and free T4 (fT4) (Gudmundsson et al., 2009).

In Mexico, the Comarca Lagunera is a region known for its extensive industrial and mining activities; as a result, the water can be contaminated with nitrates and other compounds discarded by industry. This region is the main dairy basin of northern Mexico and, consequently, there is an excessive demand to produce fodder for livestock; this has led to the indiscriminate use of pesticides and fertilizers. In addition, cattle generate a large amount of manure. The nitrogenous compounds in manure are converted to nitrates, and eventually reach water supply wells used for consumption by local communities. Therefore, the population is chronically exposed to nitrates in drinking water. In rural areas there is a high migration rate of men, therefore the population with the highest exposure are women. The aim of this study was to analyze the exposure, effect and genetic biomarkers in women with chronic exposure to nitrates in drinking water.

## Materials and Methods

### Quantification of nitrates in drinking water (wells and home)

The water collection was carried out in the period from March 2015 to December 2017. Eight communities that are supplied by the Ciudad Juárez aquifer (La Loma, Sapioriz, Ciudad Juarez, Juan E. García, Nazareno, 21 de marzo and San Jacinto) were selected for measurement of the nitrate concentration in water for human consumption. Water samples from wells (100 mL) were collected following the recommendations of the Mexican Official Guideline (NOM-127-SSA-1-1994), that establishes the permissible limits of quality and treatments that water must be submitted to, for its purification and use for human consumption. Water samples were also collected from randomly selected households located within approximately 100 m from the well that supplies the drinking water network. Determination of nitrates concentration in water samples was carried out using the selective electrode ion method with a Hoviba BW74 potentiometer. To perform the measurement, a calibration curve for potassium nitrate was generated, with a detection limit between 1 mg/L to 60 mg/L Nitrate-Nitrogen. Conversion was performed to express the results as nitrate ion.

### Study population

A cross-sectional descriptive observational study was carried out, the sampling was carried out in the period from March 2015 to December 2017. In the health centers of each community, an open call was made to invite women between 18 and 45 years old to answer a questionnaire to document general epidemiological and lifestyle data. The women had to meet the following inclusion criteria be residents of the study communities for longer than 3 years, consume water mainly from the tap or well and not to be pregnant. The study included 420 women from rural communities in the municipality of Lerdo, Durango, Mexico (21 de marzo, Sapioriz, Ciudad Juarez, San Jacinto, Juan E. García, La Loma and Nazareno). The study was approved by the Ethics Committee of the Facultad de Ciencias Químicas of the Universidad Juárez del Estado de Durango (registration number R-2014-123301538X0201-012). The study was performed under an ethical agreement and maintained individual anonymity; informed consent was obtained from each participant. The participants answered a structured questionnaire which provided information on education level, occupation, diet, personal and family pathological histories, as well as environmental and occupational exposure.

### Biological sampling

Peripheral blood samples (BD Vacutainer® serum 6 mL, BD Vacutainer® EDTA 4 mL and BD Vacutainer® lithium heparin 6 mL) and a urine sample were collected from each participant. The serum was obtained and stored at -70°C until processing biochemical parameters and thyroid hormone analyzes. Blood samples collected into the tube containing EDTA were used to measure nitrite in plasma and metHb percentage, also for DNA purification to determine *FOXE1* polymorphisms. Peripheral blood collected into the tube containing lithium heparin was used for the cytokinesis-block micronucleus cytome assay in lymphocytes. The first morning urine sample was used for nitrite determination, as an indirect biomarker of nitrate. The sample was collected in a sterile vial and stored at -70°C until processing.

### Nitrite concentration in plasma and urine

The concentrations of nitrite in plasma and urine samples were determined by the Griess colorimetric method, previously reported (Moshage et al., 1995) using spectrophotometric quantification at 540 nm. As a reference, a curve with sodium nitrite and detection limit between 0.5-30 µmol/mL was performed in triplicate and each sample was measured by duplicate. All reagents used were high purity (Sigma-Aldrich Darmstadt, Germany).

### Methemoglobin percentage

The metHb percentage was measured using the method reported by Sakata et al. (1982). Each sample was measured by duplicate. All reagents used were high purity (Sigma-Aldrich Darmstadt, Germany).

### Biochemical parameters

The biochemical parameters assessed included glucose, triglycerides, total cholesterol and HDL cholesterol which were determined following the manufacturer’s instructions (Pointe Scientific Brussels, Belgium). The LDL cholesterol concentration was calculated using the equation reported previously (Friedewald et al., 1972). The limits of the reagents were as follows: glucose, 0-500 mg/dL; cholesterol, 0-700 mg/dL; and triglycerides, 0-1000 mg/dL. The concentrations of each parameter were expressed as milligrams per deciliter.

### Thyroid-stimulating hormone, total and free T3 and T4 determinations

The quantification of thyroid hormones (TSH, total T3, fT3, total T4 and fT4) was performed by chemiluminescence immunoassays (Immulite ® 1000 Siemens Gwynedd, United Kingdom). The TSH assay had a sensitivity of 0.004 μIU/mL and an upper limit of 75 μIU/mL. The reference ranges for thyroid hormones were TSH, 0.4-4.0 μIU/mL; total T3, 82-179 ng/dL; fT3, 1-40 pg/mL; total T4, 4.5-12.5 μg/dL; and fT4, 0.3-6.0 ng/dL.

### rs965513 and rs1867277 genotyping in **FOXE1**

DNA was extracted from peripheral blood leukocytes by the standard CTAB-DTAB (Sigma-Aldrich Darmstadt, Germany) method. Two variants of the *FOXE1* gene were analyzed by real-time PCR in a Step One (Applied Biosystems, Foster City, California, USA) device using pre-designed TaqMan assays for rs965513 (C_1593670) and rs1867277 (C_11736668) (Applied Biosystems, Foster City, California, USA). The PCR assay was carried out according to the standard protocol recommended by the manufacturer.

### Cytokinesis-block micronucleus cytome assay in lymphocytes

Genotoxic damage was evaluated by a cytokinesis-block micronucleus cytome assay (Fenech, 2007). Following the culture of peripheral blood with the addition of β-cytochalasin, the preparations were stained with 5% Giemsa stain for microscopic observation. A count of 1,000 cells per individual was carried out, as suggested by the International Micronucleus Consortium; considering all binucleated cells with micronuclei, mononuclear, trinucleated and tetranucleated cells, cells with nucleoplasmic bridges and bubble protrusions, and those in necrosis and apoptosis. The proliferation index was calculated for each individual experiment. All reagents used were high purity or cell culture grade (Sigma-Aldrich Darmstadt, Germany).

### Statistical analyses

Nitrate levels in drinking water for each community were used to classify exposure as low, medium or high, based on other studies reported and the maximum permissible limit for human consumption of 50 mg/L. Data are presented as mean ± standard derivation, the variables that did not show a normal distribution are reported as median and Q1-Q3 values. To determine differences between exposure groups, Kruskal-Wallis and Dunn’s tests were applied, or a Chi-square test, depending on the variable. To determine the association between biomarkers and levels of exposure a multiple linear regression was used, adjusting for age, body mass index (BMI), consumption of alcoholic drinks, tobacco, education level and diet. All statistical analyses were performed using the STATA version 13 for Windows software package and a P value < 0.05 was considered statistically significant.

## Results

### Nitrate levels in water for human consumption in the studied communities

The general characteristics of the studied populations and nitrates concentration in water for human consumption are presented in Table 1. An increase in the concentrations reported in 2012 and 2014 was observed, with respect to the data obtained in this study. Participants were stratified in three groups according to the nitrate concentration determined in drinking water. Although there is a distinction between low, medium and high exposure, there is no consensus about the ranges and doses in each group, a strategy is to stratify according to the concentrations found in the specific study, in order to observe the exposure gradient. The average concentration of nitrates of all samples collected from wells and households were 4.7 ± 3.3, 32.1 ± 3.7 and 56.9 ± 14.7 mg/L of nitrates from low, medium and high exposure groups, respectively. Some of these values were higher than the 44.3-50 mg/L permissible limit of nitrates established by NOM-127-SSA-1-1994 and WHO (2011). The water consumption from de local network was estimated with a standardized measure (250 mL container), from this, the amount of water they drink daily was calculated. The mean values were 2.08 ± 1.03, 1.91 ± 0.82 and 2.4 ± 0.95 L for the low, medium and high exposure groups, respectively.

**Table 1 -:** Level of exposure to nitrates across time and general characteristic of the study population by communities

Levels of Nitrate mg/L by years (µ ± SD)					
**Communities**		21 marzo Sapioriz Cd. Juárez	San Jacinto Juan E. García,	La Loma Nazareno	
2010 - 2012^£^ 2013 - 2014^¥^ 2015 - 2017^§^		3.2 3.8 ± 3.2 4.7 ± 3.3	18.14 24.1 ± 3.2 32.1 ± 3.7	36.5 47.6 ± 7 56.9 ± 14.7	
Daily consumption of drinking water (L) (µ±SD)		2.08 ± 1.03	1.91 ± 0.82	2.4 ± 0.95	
**Parameter**	**All women n=420**	**Low level n=139**	**Medium level n=171**	**High level n=110**	**P-value**
Age (years)	36 (32-40)	36 (33-40)	35 (30-41)	37 (33-40)	0.4023^*^
BMI (kg/m^2^) < 24.9 25-29.9 ≥ 30	28.5 (25.5-32.4) 88 (21%) 173 (41%) 159 (38%)	29.3 (26.5-32.9) 17 (12% 62 (45%) 60 (43%)	28.5 (24.7-33.3)^a^ 45 (26%) 56 (33%) 70 (41%)	27.9 (25.3-30)^b^ 26 (24%) 55 (50%) 29 (26%)	0.0433^*^ 0.006^**^ 0.018^**^ 0.041^**^
Education < high school > high school	359 (85%) 61 (15%)	125 (90%) 14 (10%)	135 (79%) 36 (21%)	99 (90%) 11 (10%)	0.007^**^
Occupation Homemaker Seasonal agriculture	399 (95%) 21 (5%)	132 (95%) 7 (5%)	159 (93%) 12 (7%)	106 (97%) 4 (3%)	0.40^*^
Drinking water Well Purified	335 (80%) 85 (20%)	118 (85%) 21 (15%)	124 (73%) 47 (27%)	93 (85%) 17 (15%)	0.009^**^
Smoking current	28 (6%)	11 (7%)	14 (8%)	3 (2%)	0.138^**^
Alcohol consumption	57 (13%)	18 (12%)	28 (16%)	11 (10%)	0.293^**^

£  Data from Calleros Rincón *et al.*, 2012, ^¥^ Data from Conagua, ^§^ Levels measured in this study

Age and BMI (body mass index) are showed as median (Quantile 1 - Quantile 3). Stratifying of BMI, education, occupation, drinking water, smoking current and alcohol consumption are showed as n (%). Purified water by inverse osmosis method.

P-value correspond to Kruskal Wallis test (^*^) and Chi squared test (^**^), while Dunn’s test is showed with superscript letter: exposure: ^a^ low-medium*,* ^*b*^ exposure low-high.

### General characteristics of the study population

This study evaluated 420 women, of whom 139 were exposed to low levels, 171 had a medium exposure and 110 were exposed to high levels of nitrates in drinking water. For all women, the median age was 36 years (32-40 years) and median BMI was 28 (25-32). In all groups, more than 30% of the individuals were overweight or obese; over 85.5% of the participants had a basic education (primary to secondary schooling), and the predominant occupation was housewife (95%). Consumption of tap water was reported to be around 80% of all beverages, with some participants reporting infrequent consumption of bottled water from water purifying machines (purified by reverse osmosis). Consumption of alcoholic beverages was low (13.6%), and cigarette smoking (6.7%) was not frequent at either exposure level (Table 1). In addition, the diet of the study population was based on processed meat (bacon 2.8%, sausage 11% and ham 7%), vegetables (spinach 2.4%, cabbage 7%, lettuce 12%, carrots 13%, cucumber 9%, broccoli 4.8%, pepper 65%, tomato 68%) and fruits (orange 38%, lemon 29%, watermelon 9%), but there are no differences in consumption among groups.

### Alterations in biochemical parameters

The nitrite concentration was determined in plasma and urine samples and we observed a dose dependent increase (p=0.001). The percentage of metHb showed a dose dependent increase (p=0.001). The correlation between the doses of nitrate and the percentage of methemoglobin was Spearman=0.25 (p=0.0001), while the correlation between the doses of nitrate and concentration of circulating nitrite in the plasma was Spearman=0.29 (p=0.0001) and the correlation between percentage of methemoglobin and concentration of circulating nitrite in the plasma was Spearman=0.18 (p=0.0001). Finally, the correlation between concentration of circulating nitrite in the plasma and concentration of circulating nitrite in the urine was Spearman=0.38 (p=0.0001).

Among the biochemical parameters tested, the serum glucose level was increased in the high exposure group (103.2 mg/dL, p=0.001), while for the lipids, the concentration of cholesterol increased in the medium and high exposure groups (175.1 and 179.85 mg/dL, respectively, p=0.0012); HDL cholesterol decreased in the medium exposure group (49.4 mg/dL, p=0.06) and LDL cholesterol increased in the medium exposure group (98 mg/dL, p=0.001). Finally, for triglycerides the high exposure group showed the highest levels (127.7 mg/dL, p=0.001); although these were within the reference limits (Table 2).

**Table 2 -:** Biochemical parameters in women exposed to nitrates in drinking water

Parameters	All women n=420	Low n=139	Medium n=171	High n=110	Reference rank	% out of rank	P-value
Nitrite µmol/mL blood	10.37 (5.72-24.17)	7.18 (4.94-11.87)	9.15 (6.18-23.99) ^a^	24.35 (12.05-38.45) ^b,c^			0.0001^*^
Nitrite µmol/mL urine	4.09 (1.05-7.84)	2.4 (0.89-4.85)	4.45 (0.64-9.52) ^a^	5.72 (3.91-8.79) ^b^			0.0001^*^
% methemoglobin	1.94 (1.16-2.53)	1.33 (0.9-2)	1.56 (1.1-2.08) ^a^	2.7 (2.5-2.9) ^b,c^	< 1.5	80.71	0.0001^*^
Glucose mg/dL	90.35 (79.6-108.15)	86.2 (75.2-100)	88.6 (79.8-101)	103.2 (84.2-132) ^b,c^	70-110	30.94	0.0001^*^
Cholesterol mg/dL	164.85 (133.5-208.2)	151 (131-178.5)	175.1 (133.5-208.6) ^a^	179.85 (136.4-225.8) ^b,c^	< 200	29.29	0.0012^*^
HDL mg/dL	52.2 (40.4-71)	58.3 (41.5-73)	49.4 (38.5-68.6) ^a^	51.1 (41-69.4)	30-75	20.15	0.0684
LDL mg/dL	92 (74.5-132)	82.5 (66.4-104)	98 (79-135) ^a^	96 (80-144) ^b^	< 130	40.95	0.001^*^
Triglyceride mg/dL	104.4 (73.5-145.2)	101.8 (67.1-131.2)	90.1 (69.7-134)	127.7 (96-172.4) ^b^	< 150	23.57	0.0001^*^

The data are showed as median (Quantile 1 - Quantile 3).

P-value correspond to Kruskal Wallis test. * Significant differences

Significant differences in Dunn’s test (p<0.05): ^a^ exposure low-medium*,* ^*b*^ exposure low-high, ^c^ exposure medium-high

### Alterations in the thyroid hormonal profile and effect of *FOXE1* polymorphisms

**Figure 1 -:**
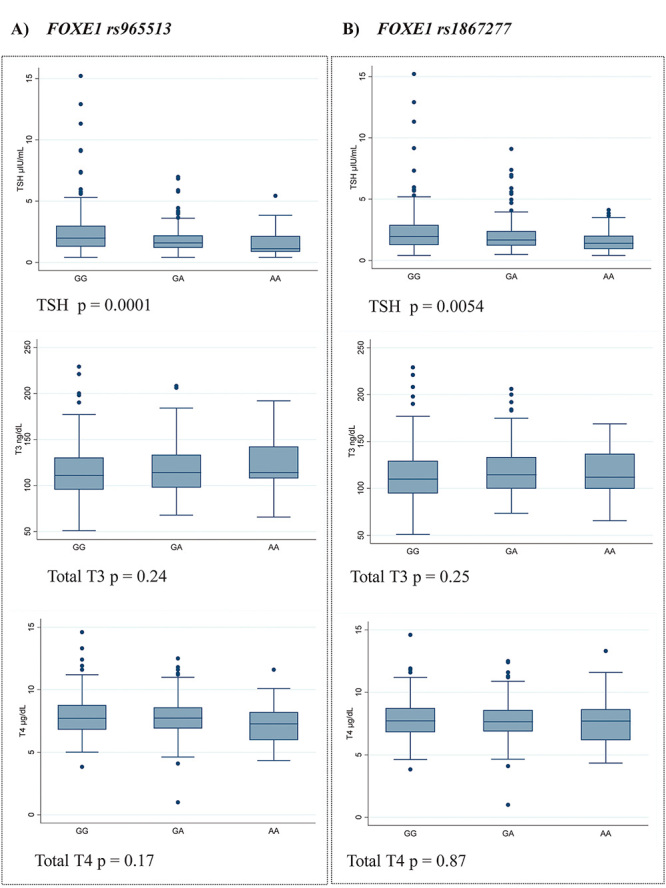
THS levels according to *FOXE1* genotypes. A) rs965513 polymorphism. B) rs1867277 polymorphism. P-values correspond to the Kruskal-Wallis test.

The serum concentrations of thyroid hormones are presented in Table 3. The TSH medians (Q1-Q3) were 1.63 (1.18-2.42), 2.02 (1.3-3.01), 1.6 (1.17-2.49) μIU/mL from low, medium and high exposure groups (p=0.01), respectively. Differences were found in the levels of fT3 (p=0.0003), T4T (p=0.01) and fT4 (p=0.0004) in the medium and high exposure groups; while the frequency of subclinical hypothyroidism (SH) did not show significant variation between groups (p=0.2). As mentioned above, *FOXE1* gene polymorphisms may cause a high risk to thyroid disorders. In the study population, the rs965513 SNP had an allelic frequency of 0.25 to polymorphic allele (A) (gMAF=0.21), and the rs1867277 SNP was found with an allelic frequency of 0.30 to polymorphic allele (A) (gMAF=0.31). Both polymorphisms were in Hardy-Weinberg equilibrium. A stratified analysis by exposure group was performed and significant differences were found in the genotypic frequencies of the rs965513 SNP and the rs1867277 SNP (Table 4). A decrease in TSH levels was also found when the polymorphic allele was in heterozygous or homozygous condition, the behavior was the same for both SNPs: rs965513 (p=0.0001) and rs1867277 (p=0.005) (Figure 1).

**Table 3 -:** Levels of thyroid hormones in women exposed to nitrates in drinking water.

Parameters	All women n=420	Low n=139	Medium n=171	High n=110	Reference rank	P-value
TSH µUI/mL	1.74 (1.24-2.67)	1.63 (1.18-2.42)	2.02 (1.3-3.01) ^a^	1.6 (1.17-2.49) ^c^	0.4-4	0.0126^*^
T3T ng/dL	113 (98.1-132)	110 (95.4-128)	115 (99-140) ^a^	113 (100-128)	82-179	0.1564
fT3 pg/mL	3.48 (3.07-4.05)	3.49 (3.17-3.92)	3.59 (3.07-4.63) ^a^	3.31 (3.05-3.67) ^b, c^	1-4	0.0003^*^
T4T µg/dL	7.7 (6.8-8.65)	7.85 (7.06-8.97)	7.46 (6.55-8.62) ^a^	7.5 (6.8-8.32) ^c^	4.5-12.5	0.0186^*^
fT4 ng/dL	1.07 (0.97-1.17)	1.08 (1-1.19)	1.03 (0.94-1.13) ^a^	1.11 (0.99-1.2) ^c^	0.3-6	0.0004^*^
Subclinical hypothyroidism	35 (8.33)	10 (7.19)	19 (11.11)	6 (5.45)		0.206§

The data are showed in median (Quantile 1 - Quantile 3).

P value correspond to Kruskal Wallis test. *Significant difference. Significant differences in Dunn’s test (p<0.05): ^a^ exposure low-medium*,* ^*b*^ exposure low-high, ^c^ exposure medium-high*.*

Subclinical hypothyroidism was considered if TSH >4 µUI/mL, n (%) are showed. § Chi squared test.

**Table 4 -:** Genotypic and allelic frequencies in women exposed to nitrates in drinking water.

Genotype	All women n=420	Low n=139	Medium n=171	High n=110	P-value
***FOXE1* rs965513**					
Genotype GG GA AA	241 (57.4%) 150 (35.7%) 29 (6.9%)	84 (60.5%) 47 (33.8%) 8 (5.7%)	107 (62.6%) 55 (32.2%) 9 (5.2%)	50 (45.5%) 48 (43.6%) 12 (10.9%)	0.042 0.43
Allele G A HWE	632 (75%) 208 (25%)	215 (77%) 63 (23%)	269 (78%) 73 (22%)	148 (67%) 72 (33%)	
***FOXE1* rs1867277**					
Genotype GG GA AA	214 (51%) 162 (38.6%) 44 (10.4%)	82 (59%) 45 (32.4%) 12 (8.6%)	99 (58%) 56 (32.7%) 16 (9.3%)	33 (30%) 61 (55.5%) 16 (14.5%)	0.0001 0.11
Allele G A HWE	590 (70%) 250 (30%)	209 (75%) 69 (25%)	254 (75%) 88 (25%)	127 (58%) 93 (42%)	

P-value corresponds to Chi squared test

HWE=Hardy-Weinberg Equilibrium

### Genotoxic damage in women with chronic exposure to nitrates

Nitrate generates RNS during its biotransformation, so in this study we evaluated the genotoxic damage present in the lymphocytes of women chronically exposed to nitrates in drinking water. The cytokinesis-block micronucleus assay was used because the cells are stimulated to enter the cell cycle and manifest damage that had not yet been observed in G0 cells. This is a technique validated by the international micronucleus consortium that allows the analysis of multiple alterations, including necrosis and apoptosis, it is also considered a cancer risk marker. In this respect, the presence of binucleated cells with micronuclei increased in the groups with medium (41.8%) and high level of exposure (42.6%), p=0.0001. These values exceed the established normal range (0-30%). Also, the number of binucleated cells with nucleoplasmic bridges and binucleated cells with bubble protrusions exceeded the normal range. Differences were significant among groups: p=0.03 and p=0.002, respectively (Table 5).

**Table 5 -:** Genotoxic damage in women exposed to nitrates in drinking water.

Parameter	All women n=420	Low n=139	Medium n=171	High n=110	Reference rank	P-value
Index of nuclear division	1.85 (1.82-1.86)	1.84 (1.81-1.86)	1.85 (1.83-1.87)	1.85 (1.78-1.87)	1.3-2.2	0.0896
Mononuclear cells %	15.5 (12.7-18.2)	16.9 (13.9-19.4)	15.8 (12.9-17.9) ^a^	13 (10.8-15.6) ^b,c^	-	0.0001^*^
Binucleated cells %	34.9 (29.7-39.4)	38.4 (34.6-40.1)	32.6 (27.3-38.4) ^a^	34.2 (26.5-39.3) ^c^	30-60	0.0001^*^
Multinucleated cells	22 (19-26)	21 (18-26)	23 (19-26) ^a^	21.5 (19-26)	-	0.0711
Apoptosis cells %	15 (11-19)	16 (11-21)	15 (12-19)	13 (11-19)	0-7	0.4990
Necrotic cells %	11 (8-16)	12 (9-16)	11 (8-16)	11 (8-18)	0-9	0.7126
Micronucleus %	38.9 (35.6-45.6)	35.2 (31.6-38.7)	41.8 (37.1-47.7) ^a^	42.6 (37.6-48.3) ^c^	0-30	0.0001^*^
Cells with bridge %	24 (19-29)	24 (19-29)	24 (19-28)	25.5 (21-31)	0-10	0.0321
Cells with bubble %	25 (19-31)	25 (19-34)	23 (18-28) ^a^	26 (21-34)	0-5	0.0021^*^

Reference Rank was based on Fenech (2007) methodology.

P-value correspond to Kruskal Wallis test. * Significant differences

Significant differences in Dunn’s test (p<0.05): ^a^ exposure low-medium*,* ^*b*^ exposure low-high, ^c^ exposure medium-high*.*

### Multivariable analysis of the association between biomarkers and concentration of nitrates in drinking water

Alterations in some biochemical and thyroid parameters were observed in the population exposed to medium and high concentrations of nitrates, therefore, a multiple regression analysis was carried out to determine the strength and magnitude of the associations. In the model, it was observed that high levels of exposure to nitrate through drinking water predicted an increase in TSH levels (β coefficient=0.21, p=0.007) and these were negatively influenced by the polymorphic allele of the rs965513 SNP (GA: β coefficient= - 0.17, p=0.01, AA: β coefficient= - 0.4, p=0.002). Also, an increase in the levels of T3 (β coefficient=0.07, p=0.008) and fT3 (β coefficient=0.12, p=0.0001) was observed; as well as a decrease of T4 (β coefficient= - 0.07, p=0.008), in the medium exposure group. In addition, significant changes were observed in the levels of nitrite in urine, as well as in the levels of glucose, total cholesterol, HDL cholesterol and triglycerides (Table 6).

**Table 6 -:** Multivariate analysis of the association between thyroid hormone, biomarkers and concentration of nitrates in drinking water.

	TSH µUI/mL			T4 ng/dL			T4 free			T3 pg/mL			T3 free		
	β	SE	p-value	β	SE	p-value	β	SE	p-value	β	SE	p-value	β	SE	P-value
Low exposure	1			1			1			1			1		
Medium exposure High exposure	0.21 -0.01	0.08 0.08	0.007^*^ 0.833	-0.07 -0.03	0.02 0.03	0.008^*^ 0.298	-0.03 -0.0007	0.02 0.02	0.070 0.972	0.07 0.02	0.03 0.03	0.008^*^ 0.447	0.12 -0.007	0.03 0.03	0.0001^*^ 0.822
Nitrite µmol/mL blood	0.02	0.03	0.500	0.002	0.01	0.843	0.006	0.008	0.433	0.001	0.01	0.882	-0.007	0.01	0.571
Nitrite µmol/mL urine	-0.03	0.02	0.180	-0.01	0.007	0.018^*^	0.013	0.005	0.012^*^	0.005	0.007	0.463	0.005	0.008	0.521
% <Methemoglobin	0.002	0.04	0.966	-0.005	0.02	0.716	-0.007	0.01	0.542	0.02	0.02	0.274	0.005	0.02	0.770
Glucose mg/dL	0.08	0.10	0.417	-0.04	0.03	0.217	-0.004	0.02	0.854	-0.09	0.03	0.007^*^	-0.07	0.04	0.080
Cholesterol mg/dL	-0.05	0.09	0.549	-0.07	0.03	0.030^*^	-0.04	0.02	0.092	-0.03	0.03	0.311	-0.05	0.03	0.125
HDL mg/dL	0.02	0.07	0.725	-0.005	0.02	0.851	0.006	0.02	0.761	0.03	0.03	0.264	0.008	0.03	0.788
LDL mg/dL	0.12	0.09	0.182	0.02	0.03	0.451	0.05	0.02	0.021^*^	0.03	0.03	0.309	-0.011	0.03	0.463
Triglyceride mg/dL	0.06	0.06	0.317	0.002	0.02	0.893	0.008	0.01	0.581	0.07	0.02	0.0001^*^	0.018	0.02	0.419
*FOXE1* rs965513 GG GA AA	1 -0.17 -0.40	0.06 0.13	0.015^*^ 0.002^*^	1 -0.03 -0.11	0.02 0.04	0.204 0.017^*^	1 -0.02 -0.03	0.01 0.03	0.258 0.301	1 0.02 0.09	0.02 0.05	0.299 0.039^*^	1 0.03 0.10	0.02 0.05	0.278 0.048^*^
*FOXE1* rs1867277 GG GA AA	1 -0.001 -0.09	0.07 0.11	0.984 0.418	1 0.002 0.01	0.02 0.04	0.904 0.751	1 0.02 0.03	0.02 0.03	0.100 0.312	1 0.02 0.03	0.02 0.04	0.241 0.418	1 0.006 -0.02	0.03 0.04	0.814 0.611
% Binucleated cells with MN	0.0002	0.0004	0.605	0.0001	0.0001	0.643	-0.0002	0.0001	0.034^*^	0.0001	0.0001	0.754	-0.0001	0.0001	0.956

TSH y nitrite were transformed to logarithmic scale.

Linear multivariate regression; adjusted by age, BMI, educational, drinking water, smoking current and alcohol consumption.

SE standard error. * Significant difference

## Discussion

The concentration of nitrates present in the study area has increased in recent years. Previously, we reported levels between 3.2-36.5 mg/L of nitrate in 2010-2012 (Calleros Rincón et al., 2012), while measurements from the National Water Commission (CONAGUA, by its acronym in Spanish), in Durango region, Mexico, reported levels between 3.8-47.6 mg/L of nitrates in 2013-2014 (https://sigagis.conagua.gob.mx/gas1/sections/Edos/durango/durango.html). For the current study we found levels ranging from 4.7-56.9 mg/L of nitrates in water for human consumption. All the data correspond to the same aquifer, indicating that there has been an increase in nitrate concentrations over the years. The increase in nitrate concentrations observed may be due to several factors, including agricultural overproduction, the lack of maintenance of the water supply network and the presence of other physical and biological factors.

The nitrate concentrations in drinking water from some communities analyzed in our study exceed the maximum permissible limit of 50 mg/L. In a study conducted in Mérida, Yucatán, Mexico, reported concentrations of 15.51-70.61 mg/L in water for human consumption. The authors studied the risk to the exposed population and found that infants have a higher risk than adults, therefore they suggested the implementation of strategies to control nitrate contamination sources and improve water quality (Rojas Fabro et al., 2015). In a review study, the presence of nitrates in water for human consumption was analyzed and it was found that the concentration of nitrates is higher in agricultural and livestock areas. These areas were designed as “nitrate vulnerable zones” and are subject to a special regulation included in the Codes of Good Agricultural Practice. The aforementioned study collects data since 1950, in different countries, and mentions that some European countries have carried out actions to recover aquifers and decrease the presence of nitrates in drinking water. However, other Asian and African countries report higher levels, between 40-100 mg/L of nitrates; and some agricultural wells show extremely high levels: up to 500 mg/L of nitrates. In these zones, levels of methemoglobinemia are monitored in the child population (Ward et al., 2018). The first effect associated with the consumption of nitrates in drinking water was methemoglobinemia in lactating children, and based on this finding, the permissible limits were determined to avoid acute effects. One of the most notable studies was the case of children in Gaza; this region contains very high levels of nitrates in the drinking water and 65.9% of children aged between 3 to 6 months had metHb > 5% and the reference level was 1.5%-2% (Abu Naser et al., 2007).

The ingested nitrate is metabolized to nitrite and NO, consequently metHb increases and the amount of oxygen available in the blood decreases; additionally, nitrite plasma levels increase, as well as its excretion in urine, with a linear relationship between the urinary nitrate excretion rate and plasma. In this study we found an increase in the percentage of metHb and nitrite in plasma and urine, and correlation between these parameters. In all cases the concentration increased according to the exposure doses. Our results agree with other studies, Bondonno et al. (2015) reported an increase in nitrite concentrations in plasma and urine as a result of exposure to nitrates; Kapil et al. (2018) found an increase in plasma nitrate after intake of dietary inorganic nitrate, the levels were higher in women than in men and were dependent on the oral microbiota. While Williams et al. (2020) found that the plasma nitrate concentration depends on the renal efficiency to excrete it to the urine; therefore, recirculation and clearance of nitrate depends on different factors. In contrast, metHb formation is constant in the organism, and to maintain a normal range (<1.5%), endogenous enzymatic systems such as NADH-dependent cytochrome b5 reductase and NADPH-dependent methemoglobin reductase are involved. It has been demonstrated that metHb values return to a basal state after 24 hours, but exposure to oxidizing agents, such as nitrate and its metabolites, accelerates the rate of metHb formation, exceeding the endogenous enzymatic systems and increasing its percentage in the blood (Thomas et al., 2019).

Regarding the biochemical parameters analyzed in this study, a significant increase was found in the concentrations of glucose, triglycerides, total, HDL and LDL cholesterol from the exposed groups. In this respect, Bahadoran et al. (2018) reported data from a 20-year cohort study, where they found an association between nitrate metabolites and the risk of developing metabolic syndrome (OR=1.75 95% CI=1.19-2.59) and type II diabetes (HR=2.43, 95% CI=1.45-4.05).

Meanwhile NO and its stable metabolites (nitrite and nitrate) have a dual role because they are related to normal and pathological processes. Furthermore, the endogenous production of NO is carried out by the isoforms of the nitric oxide synthase enzyme; however, it has not been possible to determine the endogenous and exogenous contribution of NO-nitrite-nitrate and its homeostatic balance to maintain the health of the organism. In another study, NO-nitrite-nitrate plasma levels were analyzed, and it was found that these are affected by aging, smoking habits, pregnancy, menopause, thyroid hormones and pathologies such as: type II diabetes, resistance to insulin, hypertension and kidney dysfunction (Bahadoran et al., 2019). However, it is important to determine the endogenous and exogenous contribution to the development of these pathologies. Additionally, an experimental study showed that long-term dietary nitrate and nitrite causes metabolic syndrome, endothelial dysfunction, and cardiovascular damage in mice (Kina-Tanada et al., 2017).

On the other hand, *FOXE1* is a transcription factor that regulates the expression of the thyroglobulin (TG) and thyroid peroxidase (TPO) genes; the expression of both genes is essential to the synthesis of T3 and T4 hormones, and these are necessary for maintaining a differentiated state of the thyroid gland. In addition, thyroid hormones play an important role in different organs, where they participate in cell metabolism, growth, development and differentiation. Thyroid disorders related with nitrates in drinking water have been reported (Aschebrook-Kilfoy et al., 2012) one of them is SH, that is characterized by increased TSH levels and normal T3 and T4 levels. In the present work we found 8.3% of SH in the total population, and 11% in the medium exposure group (~ 30 mg/L nitrates). In this regard, Modarelli and Ponzo (2019) reported 40.6% of SH, many of the individuals consumed water from agricultural wells, where nitrate levels ranged between 24-83 mg/L. We also found alterations in the levels of fT3, T4 and fT4, these associations are maintained in the multiple regression model and statistical significance is observed in the medium exposure group. Some authors have reported that alterations in thyroid hormones can lead to the development of thyroid cancer (Huang et al., 2017). Additionally, the rs965513 polymorphism of *FOXE1* has been associated with sporadic and familial cases of thyroid cancer. Gudmundsson et al. (2009) report that the polymorphic allele (A) is associated with low TSH concentrations, also found that T3 and T4 are also modulated by the presence of polymorphisms. Our data agrees with these findings, we found that the presence of the A allele decreases TSH concentrations; this effect was also observed in the *FOXE1* rs1867277 polymorphism, suggesting that the thyroid alterations observed in the study population may be influenced by the environment (nitrates in drinking water) and by genetic factors (polymorphisms in the *FOXE1* gene).

Finally, nitrates are metabolized to nitrite and NO, and this could generate RNS through the nitrosylation of cysteine residues in proteins, or via nitration reactions to form nitro adducts with fatty acids, proteins and nucleosides, generating genotoxic damage. Regarding the genotoxic damage in lymphocytes, we found a high percentage of binucleated cells with micronuclei, binucleated cells with nucleoplasmic bridges and binucleated cells with bubble protrusion, in medium and high exposure groups. These results agree with findings reported by Andreassi et al. (2001) they observed a higher frequency of micronucleated lymphocytes and suggest possible chromosome alterations in humans exposed to chronic long-term nitrate therapy, and van Breda et al. (2019) reported the formation of N-nitroso compounds in faecal water caused by the consumption of nitrates in drinking water, vegetables, and cured meat. Increasing the excretion of nitrate in urine and the concentration of N-nitroso compounds during the exposure time. The genotoxic damage of the metabolites was analyzed by comet assay, and an increase in DNA damage was observed in CaCo2 cells.

Finally, the effects of environmental exposure to nitrates have been reviewed previously (Edwards and Hamlin, 2018) and concluded that nitrates follow a nonmonotonic dose-response curve. Thus, it is important to understand the physiology of nitrate exposure under different scenarios as acute and subacute studies at low doses cause hormonal alterations, subchronic studies at moderate doses cause an increase in steroid hormones, and very high doses can result in cytotoxic effects; however, it is necessary to characterize the effects in different environmental conditions and in various populations. In conclusion our results showed that nitrates concentrations in drinking water are above the permissible limit and an increase in the percentage of metHb, nitrite in blood plasma and urine were found. Also, metabolic and hormonal alterations in glucose, total cholesterol, LDL, triglycerides, TSH fT3, T4T and fT4, but *FOXE1* (rs965513, rs1867277) genotypes were associated with a decrease in TSH, suggesting a gene-environment interaction. A high percentage of the samples had micronuclei in binucleated cells. Therefore, the exposure to nitrates in drinking water have a negative effect on human health in chronically exposed women.
